# Modeling and Computing of Stock Index Forecasting Based on Neural Network and Markov Chain

**DOI:** 10.1155/2014/124523

**Published:** 2014-03-23

**Authors:** Yonghui Dai, Dongmei Han, Weihui Dai

**Affiliations:** ^1^School of Information Management and Engineering, Shanghai University of Finance and Economics, 777 Guoding Road, Shanghai 200433, China; ^2^Shanghai Financial Information Technology Key Research Laboratory, 777 Guoding Road, Shanghai 200433, China; ^3^School of Management, Fudan University, 220 Handan Road, Shanghai 200433, China

## Abstract

The stock index reflects the fluctuation of the stock market. For a long time, there have been a lot of researches on the forecast of stock index. However, the traditional method is limited to achieving an ideal precision in the dynamic market due to the influences of many factors such as the economic situation, policy changes, and emergency events. Therefore, the approach based on adaptive modeling and conditional probability transfer causes the new attention of researchers. This paper presents a new forecast method by the combination of improved back-propagation (BP) neural network and Markov chain, as well as its modeling and computing technology. This method includes initial forecasting by improved BP neural network, division of Markov state region, computing of the state transition probability matrix, and the prediction adjustment. Results of the empirical study show that this method can achieve high accuracy in the stock index prediction, and it could provide a good reference for the investment in stock market.

## 1. Introduction

The stock market is filled with the coexistence of high-risk and high-yield characteristics. As a barometer of the stock market, the stock index is an important reference for investors to make investment strategies. However, the stock price index is influenced by many factors such as the economic situation, policy changes, and emergency. Although faced with complicated challenges, the forecast of stock index has still attracted the attention of many industrial experts and scholars. Lendasse et al. used a nonlinear time series model to forecast the tendency of the Bel 20 stock market index [1]. Lee et al. forecasted Korean Stock Price Index (KOSPI) by three forecasting models including back-propagation neural network model (BPNN), Bayesian Chiao's model (BC), and the seasonal autoregressive integrated moving average model (SARIMA) [2]. Fan and Gao proposed “Grey Neural Network model (GNNM(1, N))” and argued that the combined model could improve the prediction accuracy and reduce the computation [3].

Up to now, stock prediction has still been a hot topic. In this field, there have been a lot of methods, such as artificial neural networks [4, 5], time series model [6, 7], decision trees [8], Bayesian belief networks [9], evolutionary algorithms [10], fuzzy sets [11], and Markov model [12–14]. However, the single method is usually limited to achieving an ideal precision in the dynamic market due to complicated influencing factors. In recent years, some new hybrid models have shown the potential superiorities [15–17]. Especially, the approach based on adaptive modeling and conditional probability transfer may be suitable for matching the problem's characteristics.

In order to explore the new solution for improving the forecast precision, this paper presented a new method based on BP neural network and Markov chain, studied its modeling and computing technology with the data of Chinese Growth Enterprise Market, and hereafter conducted an empirical analysis of the prediction results. This paper is arranged as the following five sections: Section 1 is the introduction of research background and the most related literature; Section 2 expounds the methodology and technology as well as the combined model based on BP neural network and Markov chain; Section 3 discusses the modeling and computing technology of the presented method; Section 4 is the empirical analysis of prediction results; and the conclusion and discussion are finally in Section 5.

## 2. Methodology and Technology

### 2.1. BP Neural Network (BPNN)

BPNN is a one-way propagation of BP algorithm based on multilayer network. It is based on gradient descent method which minimizes the total of the squared errors between the actual and the desired output values. The structure of three layers BPNN includes the input layer, the hidden layer, and the output layer. The BP learning algorithm of three layers can be described as follows [18, 19].

**Step 1**

Initialize all the values of *w*_*ij*_(*t*), *w*_*jk*_(*t*), *θ*_*j*_(*t*), *θ*_*k*_(*t*) to small random values within the range [−1, 1], where *w*_*ij*_(*t*) means the connection weights between neurons *i* in the input layers and neurons *j* in the hidden layers during the *t*th learning process, *w*_*jk*_(*t*) represents the connection weights between neurons *j* in the hidden layers and neurons *k* in the output layers during the *t*th learning process, *θ*_*j*_(*t*) means the threshold value in the hidden layers, and *θ*_*k*_(*t*) means the threshold value.

**Step 2**

Select sample data and then apply the input vector *X*(*i*) = (*x*_1_, *x*_2_,…, *x*_*n*_) and desired output vector *D*(*i*) = (*d*_1_, *d*_2_,…, *d*_*n*_).

**Step 3**

Compute the outputs *y*_*j*_′ in every hidden layer, and compute the outputs *y*_*k*_ in output layer; here, *f*(·) = 1/(1 + *e* ^−*x*^) or *f*(·) = (1 − *e* ^−*x*^)/(1 + *e* ^−*x*^) is adapted activation function:

(1) yj′=f(∑i=1nwij(t)×xi−θj(t)),yk=f(∑j=1pwjk(t)×yj−θk(t)).

**Step**

Calculate the error terms *δ*_*o*_(*k*) for the output nodes:

(2) $$\begin{array}{c} \delta_{o}\left(k\right)=y_{k}\left(1-y_{k}\right)\left(d_{k}-y_{k}\right), \end{array}$$

where *d*_*k*_ represents desired output.

**Step 5**

Calculate the error terms *δ*_*j*_(*k*) for the hidden nodes:

(3) $$\begin{array}{c} \delta_{j}\left(k\right)=y_{j}^{\prime }\left(1-y_{j}^{\prime }\right)\left(\sum_{j=1}^{n}w_{jk}\delta_{o}\left(k\right)\right). \end{array}$$

**Step 6**

Update weights on the output layer:

(4) $$\begin{array}{c} w_{jk}\left(n\right)=w_{jk}\left(n-1\right)+\Delta w_{jk}\left(n\right), \end{array}$$

where

(5) Δwjk(n)=−η∂e∂wjk+αΔwjk(n−1)=ηδo(k)yk+αΔwjk(n−1).

**Step 7**

Update weights on the hidden layer:

(6) $$\begin{array}{c} w_{ij}\left(n\right)=w_{ij}\left(n-1\right)+\Delta w_{ij}\left(n\right), \end{array}$$

where

(7) Δwij(n)=−η∂e∂wij+αΔwij(n−1)=ηδj(k)xi+αΔwij(n−1).

**Step 8**

Calculate error; repeat Steps  2–8 until the error falls below a predefined threshold:

(8) $$\begin{array}{c} E_{p}=\frac{1}{2}\sum_{j=1}^{m}{\left(d_{k}-y_{k}\right)}^{2}, \end{array}$$

where *m* means the number of output node.

Although BP algorithm is successful, it has some disadvantages such as lower convergence speed and easy to get into local minima points. Therefore, improved BP algorithm was applied in our study. Our improved method is based on the additional momentum and adaptive learning rate combined. The formula with the momentum factor weight adjusting is as follows:

(9) $$\begin{array}{c} \Delta w\left(k+1\right)=\text{lr}\times \text{mc}\times \frac{\partial E}{\partial w}+\text{mc}\times \Delta w\left(k\right), \end{array}$$

where *w* represents network weight, *k* is number of training, lr is the learning rate, mc is the momentum coefficient, 0 < mc < 1, and *E* is the error function.

In addition, adaptive learning rate method can be described as follows:

(10) $$\begin{array}{c} \text{lr}\left(k+1\right)=\{\begin{array}{cc} \alpha \times \text{lr}\left(k\right) & \text{if}\;\;E\left(i+1\right)<E\left(i\right)\\ \beta \times \text{lr}\left(k\right) & \text{if}\;\;E\left(i+1\right)>\gamma \times E\left(i\right)\\ \text{lr}\left(k\right) & \text{otherwise}, \end{array} \end{array}$$

where lr is the learning rate, *k* is the number of training, *E* is the error function, $E={\sum_{i=1}^{n}\left(y_{i}-{\hat{y}}_{i}\right)}^{2}$, *y*_*i*_ is actual output value, and ${\hat{y}}_{i}$ is anticipative output value; usually *α* = 1.05, *β* = 0.7, and *γ* = 1.04 [20].

### 2.2. Markov Chain

Discrete-time Markov chain can be described as a sequence of random variables {*X*(*t*), *t* ∈ *T*}, where *T* = {1,2,…, *N*} and state space *S* = {1,2,…, *M*}. For any time *n* ≥ 0 and any state (*i*_0_, *i*_1_,…, *i*_*n*−1_, *i*, *j*) ∈ *S* and positive integer step *k*, when this sequence of variables has the following attributes:

(11) P{X(n+k)=in+k ∣ X(n)=in,X(n−1)=in−1,…,X(j2)=ij2,X(j1)=ij1}   =P{X(n+k)=in+kX(n)=in},

(12) $$\begin{array}{c} P\left\{X\left(n+1\right)=jX\left(n\right)=i\right\}=p_{ij}. \end{array}$$

We call such stochastic variable sequence {*X*(*t*), *t* ∈ *T*} Markov chains, where *p*_*ij*_ is the transition probability from state *i* to state *j*. These transition probabilities satisfied ∑_*j*∈*s*_ *p*_*ij*_ = 1, *i* ∈ *S*, and the matrix *P* = (*p*_*ij*_) is the transition matrix of the chain. If the transition probabilities in (12) do not depend on the time parameter *n*, it will be called “time-homogeneous Markov chains.”

Since the state space *S* is countable, we can label the states by integers, such as *S* = {0,1, 2,…}. Under this label, the transition matrix can be described as follows:

(13) $$\begin{array}{c} P\left(n\right)=P\left(0\right)P_{n}=P\left(0\right){\left[\begin{array}{cccc} p_{11} & p_{12} & \ldots & p_{1n}\\ p_{21} & p_{22} & \ldots & p_{2n}\\ \ldots & \ldots & & \ldots \\ p_{n1} & p_{n2} & \ldots & p_{nn} \end{array}\right]}^{n}. \end{array}$$

### 2.3. Modeling of Forecast Based on Improved BPNN and Markov Chain

The modeling process can be described as follows.

**Step 1**

Construct improved BPNN model.

**Step 2**

Initialize forecasting by using model of Step  1.

**Step 3**

Normalize the error of prediction. The normalized formula is as follows:

(14) $$\begin{array}{c} \hat{x}=\frac{x-x_{\min}}{x_{\max}-x_{\min}}. \end{array}$$

**Step**

Set Markov state zoning by normalized upper and lower thresholds.

**Step 5**

Divide the Markov state region by using the sample average-mean square deviation method. Five ranges are divided as follows [21]: $\left(-\infty ,\overset{-}{x}-\alpha_{1}s\right)$, $\left(\overset{-}{x}-\alpha_{1}s,\overset{-}{x}-\alpha_{2}s\right)$, $\left(\overset{-}{x}-\alpha_{2}s,\overset{-}{x}+\alpha_{3}s\right)$, $\left(\overset{-}{x}+\alpha_{3}s,\overset{-}{x}+\alpha_{4}s\right)$, and $\left(\overset{-}{x}+\alpha_{4}s,+\infty \right)$, where $\overset{-}{x}$ means average and *s* is sample standard deviation; usually *α*_1_ and *α*_4_ are range [1.0, 1.5] and *α*_2_ and *α*_3_ are range [0.3, 0.6].

**Step 6**

Define the initial state and calculate the state transition probability matrix.

**Step 7**

Markov chain test: use chi-square statistics test for Markov property.

**Step 8**

Forecast. Get the state vector of *k* step from formula (13) and forecast based on this model.

## 3. Modeling and Computing

### 3.1. Sample Data

In this paper, we select “Chinese Growth Enterprise Market Index (GEMI, 399006.SZ)” for the data set to empirical study, and then we will finish short-term Chinese GEM index price prediction based on this data set. The data set is total of 58 days, which is from 2013-5-24 to 2013-8-16 of trading data. Among them, divided into in-sample and out-of-sample, the first 41 days of data are in-sample as training data and then the data from 42 days are out-of-sample and used as prediction. Due to closing index price, the most important indicator for investment reference, our study focuses on the closing index price forecasting. The daily trading data including opening price, highest price, lowest price, closing price, and trading volume are used for modeling. The sample data of Chinese GEM index are shown in Table 1.

### 3.2. Modeling

#### 3.2.1. Construct BP Neural Network Model

*(i) Definition of Layer Number.* According to Kolmogorov theorem, three layers can approach any continual function. Therefore, an input layer, a hidden layer, and an output layer are selected in this model.

*(ii) Activation Function and Training Target. *Here, the activation function of hidden layer neuron is tansig, the output layer neurons traditional function is purelin.

The training function is traingdx.

The end of training conditions is the mean square error of the accuracy of *E* = 0.005.

The circulation is 10000 times.

In this model, the initial learning rate is 0.1.

The initial momentum factor value is 0.9.

*(iii) Number of Neural Node. *The input layer node number is five, namely, items of opening price, highest price, lowest price, closing price, and trading volume. Meanwhile, data of “day 1” were regarded as the first input data in input layer.

In this model, the output layer node number is set to one; meanwhile, “closing price” of “day 2” was regarded as the first output data in output layer.

Numbers of hidden layer node depend on experience and repeated training, how many of the nodes depend on the network error; the number corresponding to the minimum network error in training will be chosen as the number of the hidden layer nodes.

The network errors which correspond to different number of neurons are shown in Table 2. It can be seen that this neural network has the minimum network error of 0.2689 when the neuron number is eleven. Therefore, we select eleven as the number of hidden layer nodes. The data in Table 2 indicate that network error cannot be reduced even if we contiune to increase the number of hidden layer nodes.

#### 3.2.2. Training

**Figure 1:**
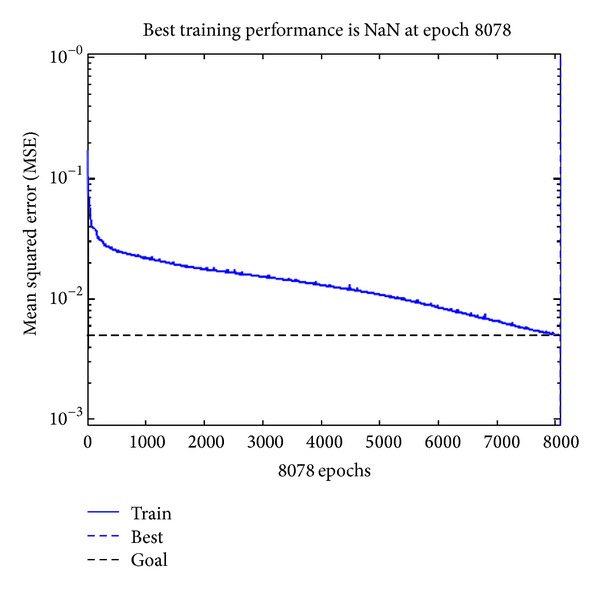
The dependence of MSE on epochs.

Training of the network is completed in MATLAB software. First, the training sample data is selected; then, the data is normalized. Normalization means to limit the data in a certain interval. Here, in order to limit training data in [−1,1], the premnmx(·) function is called. After normalization, start training network with a training set of 41 sample data; the learning rate is 0.1 and the momentum is 0.9. The network was in training till the Mean Squared Error (MSE) was less than 0.005. Finally, we get the ideal model after training the neural network. The dependence of MSE on epochs is shown in Figure 1.

It can be seen from Figure 1 that the network MSE reaches the expected MSE after 8078 steps of training, in which the training MSE is less than 0.005.

#### 3.2.3. Forecast

*(i) Initial Forecasting Based on Improved BPNN.* According to trained network and sample data, we used rolling forecasting method to predict the closing index price. Part of the code in MATLAB software is shown in Algorithm 1.

**Figure 2:**
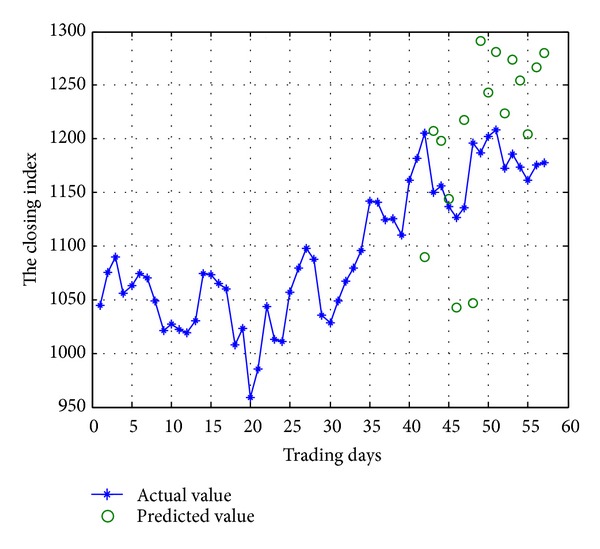
Simulation of actual value and predicted value.

The Chinese GEM index of daily closing price of simulation is shown in Figure 2. Both actual value and predicted value are shown when trading day from 42 days to 56 days.

*(ii) Computing of Normalization*

**Step 1**

Calculate the absolute residual rate of prediction days. The calculation formula is as follows:

(15) $$\begin{array}{c} y_{i}=\frac{x_{a}-x_{p}}{x_{a}}\times 100\%, \end{array}$$

where *x*_*a*_ is the actual value of closing index price, *x*_*p*_ is the predicted value of closing index price, and *y*_*i*_ is the absolute residual rate of *i* day.

**Step 2**

Normalize the data set of absolute residual rate in MATLAB software; the mapminmax(·) function is called. The absolute residual rate and normalized results are shown in Table 3.

#### 3.2.4. Empirical Markov Model

*(i) State Definition. *According to the normalization value of Table 3, sample average-mean square deviation was used in state classification. Usually five intervals are divided: $\left(-\infty ,\overset{-}{x}-\alpha_{1}S\right)$, $\left(\overset{-}{x}-\alpha_{1}S,\overset{-}{x}-\alpha_{2}S\right)$, $\left(\overset{-}{x}-\alpha_{2}S,\overset{-}{x}+\alpha_{3}S\right)$, $\left(\overset{-}{x}+\alpha_{3}S,\overset{-}{x}+\alpha_{4}S\right)$, and $\left(\overset{-}{x}+\alpha_{4}S,+\infty \right)$, where $\overset{-}{x}$ is average, *S* is sample standard deviation, *α*_1_ and *α*_4_ belong to range [1.0, 1.5], and *α*_2_ and *α*_3_ belong to range [0.3, 0.6].

Taking into account the fact that the data is not that much, Markov state was divided into four ranges according to $\left(-\infty ,\overset{-}{x}-0.4S\right)$, $\left(\overset{-}{x}-0.4S,\overset{-}{x}+0.6S\right)$, $\left(\overset{-}{x}+0.6S,\overset{-}{x}+1.5S\right)$, and $\left(\overset{-}{x}+1.5S,+\infty \right)$; therefore, Markov state ranges are (1) [0,0.1926], (2) (0.1926,0.4840], (3) (0.4840,0.7462], and (4) (0.7462,1]. Then, Markov state transition was built as shown in Table 4.

*(ii) Computing of State Transition Probability Matrix.* It can be seen from Table 4 that from the state (1) to (1) it has 2 times, from the state (1) to (2) it has 3 times, from the state (1) to (3) it has 0 times, and from the state (1) to (4) it has 0 times; then, the sate transition probability can be calculated as follows:

(16) $$\begin{array}{c} p_{11}=\frac{2}{5}=0.4,\;\;p_{12}=\frac{3}{5}=0.6,\;\\ p_{13}=\frac{0}{5}=0.0,\;\;p_{14}=\frac{0}{5}=0.0. \end{array}$$

Similarly,

(17) $$\begin{array}{c} p_{21}=\frac{3}{7}=0.4286,\;\;p_{22}=\frac{2}{7}=0.2857,\\ p_{23}=\frac{0}{7}=0.0,\;\;p_{24}=\frac{2}{7}=0.2857,\\ p_{31}=\frac{0}{1}=0.0,\;\;p_{32}=\frac{1}{1}=1.0,\\ p_{33}=\frac{0}{1}=0.0,\;\;p_{34}=\frac{0}{1}=0.0,\\ p_{41}=\frac{1}{2}=0.5,\;\;p_{42}=\frac{1}{2}=0.5,\\ p_{43}=\frac{0}{2}=0.0,\;\;p_{44}=\frac{0}{2}=0.0. \end{array}$$

Thus, the state transition probability matrix *p* can be described as follows:

(18) $$\begin{array}{c} p=\left[\begin{array}{cccc} 0.4 & 0.6 & 0.0 & 0.0\\ 0.4286 & 0.2857 & 0.0 & 0.2857\\ 0.0 & 1.0 & 0.0 & 0.0\\ 0.5 & 0.5 & 0.0 & 0.0 \end{array}\right]. \end{array}$$

The probability matrix *p* has the Markov property after chi-square statistics test.

*(iii) The Step State Vector of Prediction.* According to the state transition probability matrix *p* and the Markov forecast model, the step state vector of prediction can be calculated as follows:

(19) $$\begin{array}{c} P\left(n\right)=\left[0,0,1,0\right]\times p^{n}. \end{array}$$

Thus, the step state vector of prediction can be described as shown in Table 5.

## 4. Empirical Analysis

According to the step state vector of prediction of Markov model, prediction result from 2013-7-25 to 2013-8-15 was shown in Table 6. Among them, *V*_col6_ is adjustment value and $V_{\text{col}6}=P_{\max5}\wedge {\overset{-}{X}}_{\text{col}4}$, where *P*_max⁡5_ means the maximum probability of someday in fifth column and ${\overset{-}{X}}_{\text{col}4}$ means the average of interval of fourth column.

It can be seen from column “error of absolute residual rate” in Table 6, during sixteen trading days, that most of the prediction results by this model are better than a single improved neural network prediction except during the first day and the fifth day.

## 5. Conclusion and Discussion

Due to the complicated influencing factors in dynamic stock market, the comprehensive method with hybrid models throws off more superiorities than a single method in the forecast of stock index. This paper presented a new method based on the combination of improved back-propagation (BP) neural network and Markov chain, which took the advantages of neural network and Markov model, and obtained the results better than that of the single improved BPNN method. This method could provide a good reference for the investment in stock market.

As an open complex adaptive system constantly affected by all kinds of emergency events and people's psychological and behavioral effects, although many scholars including the famous financial experts pointed out that the changes of stock market cannot be predicted, we had to break those traditional ideas which rely only on the financial theory models and explore new combined methods such as the TDF (Theory-Data-Feedback) modeling and analyzing framework [22] and the spread model of emotions and behaviors caused by emergency events [23]. We believe that the change of the stock market has also its characteristics and inherent rules, and the forecast is possible at least in the short-term prediction.

## Figures and Tables

**Algorithm 1:**
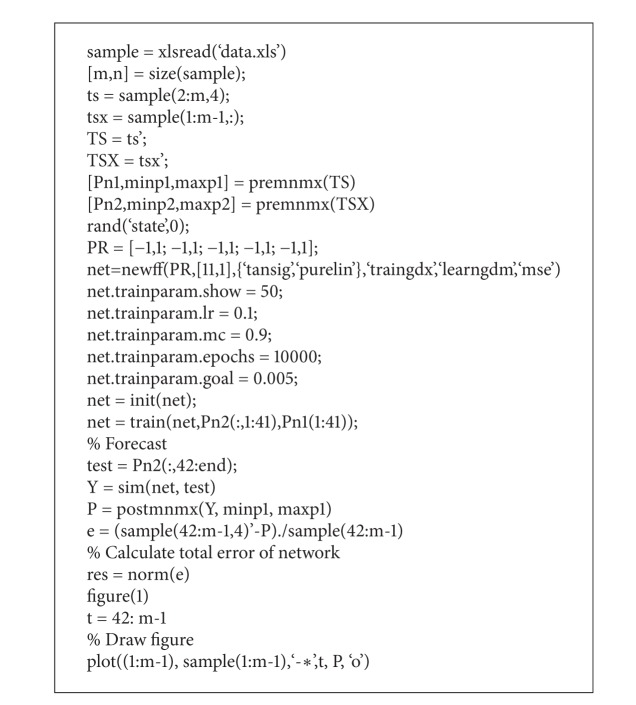
Part of the code of MATLAB.

**Table 1:** Sample data.

Days	1	2	3	4	5	6	7	8	9	10
Opening price	1044.29	1075.83	1089.77	1055.60	1063.00	1074.48	1070.73	1049.10	1021.02	1027.05
Highest price	1073.78	1091.25	1090.16	1067.03	1078.46	1086.33	1074.82	1049.26	1038.03	1031.36
Lowest price	1044.29	1075.83	1055.64	1053.18	1059.83	1068.07	1050.21	1011.40	1021.02	1020.83
Closing price	1073.78	1089.72	1059.00	1065.96	1073.87	1073.02	1052.14	1022.46	1032.76	1022.13
Trading volume	15023082	16132262	15413445	11569457	12702507	14032823	12668299	12745388	9397943	9843383

Days	11	12	13	14	15	*⋯*	55	56	57	58

Opening price	1022.56	1019.47	1029.93	1074.53	1073.31	*⋯*	1161.49	1175.63	1177.85	1158.96
Highest price	1039.65	1029.28	1069.81	1091.91	1078.53	*⋯*	1180.61	1186.29	1184.40	1178.84
Lowest price	1018.45	986.05	1029.93	1068.61	1053.33	*⋯*	1151.68	1160.02	1165.77	1131.44
Closing price	1030.14	1029.28	1068.15	1073.69	1072.93	*⋯*	1172.33	1180.66	1167.09	1132.09
Trading volume	10557897	9936156	11803687	13475017	11576013	*⋯*	13353812	16202467	15192214	15946678

Data source: the above data are from Wind information database.

**Table 2:** Error of repeated training.

Number of neuron	9	10	11	12	13	14
Network error	1,8710	0.9392	0.2689	0.5924	0.3357	0.7099

**Table 3:** Normalization of absolute residual rate.

Days	Actual value	Prediction value	Error of absolute residual rate	Normalization value
1	1148.81	1089.72	5.27%	0.6797
2	1133.16	1207.61	−4.25%	0.2457
3	1136.40	1198.35	−5.40%	0.1978
4	1095.65	1143.73	−1.75%	0.3648
5	1117.34	1043.10	8.02%	0.8214
6	1135.45	1217.55	−2.31%	0.3326
7	1187.31	1046.42	11.98%	1.0000
8	1182.54	1291.15	−7.34%	0.0985
9	1200.69	1242.76	−2.56%	0.3251
10	1173.26	1280.88	−8.95%	0.0397
11	1165.32	1223.61	−3.17%	0.2956
12	1153.45	1273.66	−8.63%	0.0478
13	1146.02	1254.19	−7.95%	0.0789
14	1151.68	1204.72	−2.76%	0.3153
15	1160.02	1266.82	−7.30%	0.1037
16	1165.77	1279.64	−9.64%	0.0000

**Table 4:** Markov state transition.

State	(1)	(2)	(3)	(4)	Total
(1)	2	3	0	0	5
(2)	3	2	0	2	7
(3)	0	1	0	0	1
(4)	1	1	0	0	2
Total	**6**	**7**	**0**	**2**	**15**

**Table 5:** Probability of step state vector.

State	Step 1	Step 2	Step 3	Step 4
(1)	0.0000	0.4286	0.4367	0.4219
(2)	1.0000	0.2857	0.4816	0.4405
(3)	0.0000	0.0000	0.0000	0.0000
(4)	0.0000	0.2857	0.0816	0.1376

State	Step 5	Step 6	Step 7	Step 8

(1)	0.4264	0.4254	0.4256	0.4255
(2)	0.4478	0.4467	0.4468	0.4468
(3)	0.0000	0.0000	0.0000	0.0000
(4)	0.1258	0.1279	0.1276	0.1277

State	Step 9	Step 10	Step 11	Step 12

(1)	0.4255	0.4255	0.4255	0.4255
(2)	0.4468	0.4468	0.4468	0.4468
(3)	0.0000	0.0000	0.0000	0.0000
(4)	0.1277	0.1277	0.1277	0.1277

State	Step 13	Step 14	Step 15	Step 16

(1)	0.4255	0.4255	0.4255	0.4255
(2)	0.4468	0.4468	0.4468	0.4468
(3)	0.0000	0.0000	0.0000	0.0000
(4)	0.1277	0.1277	0.1277	0.1277

**Table 6:** Prediction result.

Days	Actual value	Value of improved BPNN forecast	Markov prediction interval	Probability	Adjustment value	Error of absolute residual rate (improved BPNN)	Error of absolute residual rate (adjustment)
(1)	(2)	(3)	(4)	(5)	(6)	(7)	(8)
1	1150.39	1089.72	[985.6, 1030.6] [1030.6, 1098.8] [1098.8, 1160.1] [1160.1, 1219.5]	0.0000 1.0000 0.0000 0.0000	1064.72	5.27%	7.45%

2	1158.35	1207.61	[1092.2, 1142.1] [1142.1, 1217.7] [1217.7, 1285.6] [1285.6, 1351.4]	0.4286 0.2857 0.0000 0.2857	1117.18	−4.25%	3.55%

3	1136.98	1198.35	[1083.8, 1133.3] [1133.3, 1208.3] [1208.3, 1275.7] [1275.7, 1341.0]	0.4367 0.4816 0.0000 0.0816	1170.85	−5.40%	−2.98%

4	1124.10	1143.73	[1034.4, 1081.7] [1081.7, 1153.2] [1153.2, 1217.6] [1217.6, 1279.9]	0.4219 0.4405 0.0000 0.1376	1117.48	−1.75%	0.59%

5	1134.02	1043.10	[943.4, 986.5] [986.5, 1051.8] [1051.8, 1110.5] [1110.5, 1167.3]	0.4263 0.4478 0.0000 0.1258	1019.17	8.02%	10.13%

6	1190.11	1217.55	[1101.2, 1151.5] [1151.5, 1227.7] [1227.7, 1296.2] [1296.2, 1362.5]	0.4254 0.4467 0.0000 0.1279	1189.61	−2.31%	0.04%

7	1188.85	1046.42	[946.4, 989.7] [989.7, 1055.1] [1055.1, 1114.0] [1114.0, 1171.0]	0.4256 0.4468 0.0000 0.1276	1084.58	11.98%	8.77%

8	1202.83	1291.15	[1167.8, 1221.1] [1221.1, 1301.9] [1301.9, 1374.6] [1374.6, 1444.9]	0.4255 0.4468 0.0000 0.1277	1261.51	−7.34%	−4.88%

9	1211.78	1242.76	[1124.0, 1175.4] [1175.4, 1253.1] [1253.1, 1323.1] [1323.1, 1390.7]	0.4255 0.4468 0.0000 0.1277	1214.24	−2.56%	−0.20%

10	1175.70	1280.88	[1158.5, 1211.4] [1211.4, 1291.5] [1291.5, 1363.6] [1363.6, 1433.4]	0.4255 0.4468 0.0000 0.1277	1251.48	−8.95%	−6.45%

11	1185.96	1223.61	[1106.7, 1157.2] [1157.2, 1233.8] [1233.8, 1302.7] [1302.7, 1369.3]	0.4255 0.4468 0.0000 0.1277	1195.53	−3.17%	−0.81%

12	1172.52	1273.66	[1151.9, 1204.6] [1204.6, 1284.2] [1284.2, 1355.9] [1355.9, 1425.3]	0.4255 0.4468 0.0000 0.1277	1244.43	−8.63%	−6.13%

13	1161.87	1254.19	[1134.3, 1186.1] [1186.1, 1264.6] [1264.6, 1335.2] [1335.2, 1403.5]	0.4255 0.4468 0.0000 0.1277	1225.40	−7.95%	−5.47%

14	1172.33	1204.72	[1089.6, 1139.4] [1139.4, 1214.7] [1214.7, 1282.5] [1282.5, 1348.1]	0.4255 0.4468 0.0000 0.1277	1177.07	−2.76%	−0.40%

15	1180.66	1266.82	[1145.7, 1198.1] [1198.1, 1277.3] [1277.3, 1348.6] [1348.6, 1417.6]	0.4255 0.4468 0.0000 0.1277	1237.75	−7.30%	−4.84%

16	1167.09	1279.64	[1157.3, 1210.2] [1210.2, 1290.2] [1290.2, 1362.3] [1362.3, 1432.0]	0.4255 0.4468 0.0000 0.1277	1250.28	−9.64%	−7.13%

## References

[1] Lendasse A, de Bodt E, Wertz V, Verleysen M (2000). Non-linear financial time series forecasting-application to the Bel 20 stock market index. *European Journal of Economic and Social Systems*.

[2] Lee KJ, Chi AY, Yoo S, Jin JJ (2008). Forecasting Korean stock price index (kospi) using back propagation neural network model, Bayesian Chiao's model, and ASRIMA model. *Academy of Information and Management Sciences Journal*.

[3] Fan Y, Gao F A stock index forecasting model based on grey relation theory and GNNM (1, N).

[4] Li CW, Zhang J (2006). ANN-based mid-term stock forecasting. *Computer Engineering & Science*.

[5] Hanias M, Curtis P, Thalassinos E (2012). Time series prediction with neural networks for the athens stock exchange indicator. *European Research Studies Journal*.

[6] Liu W, Morley B (2009). Volatility forecasting in the hang seng index using the GARCH approach. *Asia-Pacific Financial Markets*.

[7] Srinivasan P (2011). Modeling and forecasting the stock market volatility of S&P 500 index using GARCH models. *The IUP Journal of Behavioral Finance*.

[8] Nair BB, Mohandas VP, Sakthivel NR (2010). A decision tree—rough set hybrid system for stock market trend prediction. *International Journal of Computer Applications*.

[9] Ying J, Kuo L, Seow GS (2005). Forecasting stock prices using a hierarchical Bayesian approach. *Journal of Forecasting*.

[10] Kaboudan MA (2000). Genetic programming prediction of stock prices. *Computational Economics*.

[11] Hwang H, Oh J (2010). Fuzzy models for predicting time series stock price index. *International Journal of Control, Automation and Systems*.

[12] Alizadeh A, Nomikos N (2004). A markov regime switching approach for hedging stock indices. *Journal of Futures Markets*.

[13] Li S, Hui X (2011). A new stock index fuzzy stochastic prediction model developed by introducing a Markov chain. *Journal of Harbin Engineering University*.

[14] Gong J, Ma CH (2012). A hidden Markov chain modeling of shanghai stock index. *Finance*.

[15] Kim MJ, Han I, Lee KC (2004). Hybrid knowledge integration using the fuzzy genetic algorithm: prediction of the Korea stock price index. *Intelligent Systems in Accounting, Finance and Management*.

[16] Pai P-F, Lin C-S (2005). A hybrid ARIMA and support vector machines model in stock price forecasting. *Omega*.

[17] Hassan MR, Nath B, Kirley M (2007). A fusion model of HMM, ANN and GA for stock market forecasting. *Expert Systems with Applications*.

[18] Rumelhart DE, Hinton GE, Williams RJ (1986). Learning representations by back-propagating errors. *Nature*.

[19] Yang JP (2008). *The Research of Improved BP Algorithm Based on Self-Adaptive Learning Rate*.

[20] Su GL, Deng FP (2003). On the improving back propagation algorithms of the neural networks based on MATLAB language: a review. *Bulletin of Science and Technology*.

[21] He MM (2008). *The Application on Some Economic Prediction With Markov Chain Model*.

[22] Dai WH (2014). The public cognitive mechanism of emotion on city emergency events and coping strategy. *Urban Management*.

[23] Dai WH, Wan XQ, Liu XY (2011). Emergency event: internet spread, psychological impacts and emergency management. *Journal of Computers*.

